# A Multisite Non-Inferiority Randomized Controlled Trial of the Efficacy of Cognitive-Behavior Therapy for Generalized Anxiety Disorder Delivered by Videoconference

**DOI:** 10.3390/jcm11195924

**Published:** 2022-10-07

**Authors:** Stéphane Bouchard, Michel J. Dugas, Geneviève Belleville, Frédéric Langlois, Patrick Gosselin, Geneviève Robillard, Giulia Corno, André Marchand

**Affiliations:** 1 Département de Psychologie et de Psychoéducation, Université du Québec en Outaouais, Gatineau, QC J8X 3X7, Canada; michel.dugas@uqo.ca (M.J.D.); g.robillard@invirtuo.com (G.R.); giulia.me.corno@gmail.com (G.C.); 2 Centre de Recherche du Centre de Santé et des Services Sociaux de l’Outaouais, Gatineau, QC J8T 4J3, Canada; 3 École de Psychologie, Université Laval, Québec, QC G1V 0A6, Canada; genevieve.belleville@psy.ulaval.ca; 4 Département de Psychologie, Université du Québec à Trois-Rivières, Trois-Rivières, QC G8Z 4M3, Canada; frederic.langlois@uqtr.ca; 5 Département de Psychologie, Université de Sherbrooke, Sherbrooke, QC J1K 2R1, Canada; patrick.gosselin@usherbrooke.ca; 6 Département de Psychologie, Université du Québec à Montréal, Montréal, QC H2L 2C4, Canada; marchand.andre@uqam.ca

**Keywords:** videoconferencing psychotherapy, generalized anxiety disorder, cognitive-behavior therapy, non-inferiority, predictors of outcome

## Abstract

Delivering psychotherapy by videoconference has been studied in a number of clinical trials, but no large controlled trial has involved generalized anxiety disorder (GAD). This multicenter randomized controlled non-inferiority trial was conducted to test if cognitive-behavior psychotherapy delivered by videoconference (VCP) is as effective as cognitive-behavior psychotherapy delivered face-to-face, using a strict margin of tolerance for non-inferiority. A total of 148 adults received a 15-session weekly manualized program. The treatment was based on the intolerance of uncertainty model of GAD. The impact of treatment was assessed using primary (GAD severity), secondary (worry, anxiety, and intolerance of uncertainty) and tertiary (general functioning) variables measured before and after treatment and at 6-month and 12-month follow-ups. Results showed that: (a) the treatment was effective; (b) VCP for GAD was statistically non-inferior to face-to-face psychotherapy on primary, secondary and tertiary measures at all assessment points; (c) change in intolerance of uncertainty significantly predicted change in the primary outcome measure over and above important clinical factors common to all psychotherapies (motivation, working alliance, perceived therapist competence, and client satisfaction). These findings support the use of VCP as a promising treatment option for adults with GAD. Clinical trial registry: ISRCTN#12662027.

## 1. Introduction

General anxiety disorder (GAD) is a highly common and chronic mental disorder with an estimated lifetime prevalence of 9% and a low rate of spontaneous remission [1,2,3]. Individuals with GAD experience excessive and uncontrollable anxiety and worry about different events or activities in key spheres of life such as work, health, finances, or family [1]. The prevalence and severity of this disorder are comparable in rural and urban zones, as are its psychosocial characteristics and comorbidity profile [4,5]. Individuals with GAD report high levels of psychological distress, low quality of life and high impairment at work, with high rates of absenteeism and low productivity [6]. Furthermore, patients with GAD are frequent users of healthcare services, leading to major costs to the healthcare system [7].

Cognitive-behavior therapy (CBT), which is considered to be the gold standard therapy for anxiety disorders, is recommended as the first-line treatment for GAD. Indeed, its efficacy and effectiveness for GAD has been extensively demonstrated [8,9,10,11]. The conceptual model of GAD proposed by Dugas and his colleagues [12,13] rests on the key role of intolerance of uncertainty (a negative dispositional characteristic arising from a set of catastrophic beliefs about uncertainty and its consequences). Intolerance of uncertainty is theorized to be a key mechanism involved in positive beliefs about worry, cognitive avoidance and chronic anxiety [12,13,14]. Based on this model, Dugas and colleagues developed a cognitive-behavioral treatment for GAD that is focused on intolerance of uncertainty (IU) and is now commonly referred to as CBT-IU [15]. Several randomized controlled trials have demonstrated the efficacy of CBT-IU for GAD [16,17,18,19,20,21]. 

Many individuals, including those with GAD, do not have access to specialized mental health services [22]. People in rural areas can face significant challenges in finding and attending face-to-face psychological services with mental health professionals who often practice in urban areas [5,22,23,24]. Even in urban areas, it can be difficult to regularly attend psychotherapy sessions because of other barriers to service utilization [24] such as structural barriers (e.g., commuting to the therapist’s office), availability of professionals sharing the patient’s cultural or ethnic values, or availability of psychotherapists with a specific expertise. The COVID-19 pandemic increased barriers to face-to-face treatment [25] due to recommendations regarding confinement, physical distancing, wearing surgical masks during consultations, etc.

Although telepsychology has traditionally been considered as a solution for providing access to mental health services for people living in rural areas, it is now considered to be a viable alternative to face-to-face psychotherapy [26,27,28,29,30,31]. Indeed, an increasing number of studies have documented the efficacy and effectiveness of videoconferencing psychotherapy (VCP) for mental health disorders [26,29,30,32,33]. Moreover, a handful of studies have documented the non-inferiority of VCP when compared to gold-standard treatments [34,35,36,37,38]. All reviews have highlighted the need for more randomized controlled trials for mental health disorders that have not yet been studied [26,29,38], such as GAD.

Only two studies have investigated the potential effectiveness of VCP specifically for patients with GAD. In a first uncontrolled study, Griffiths, Blignault and Yellowlees [39] provided CBT to 15 adults suffering from a variety of disorders, including three diagnosed with GAD. Although the overall results showed statistically significant pre to post-treatment differences on measures of anxiety and depression, the authors did not report the specific findings for the participants diagnosed with GAD. Théberge-Lapointe et al. [40] provided CBT-IU to five adults diagnosed with GAD using a multiple baseline design across participants. Their results showed preliminary support for the efficacy of VCP for GAD. Participants’ anxiety improved at post-treatment, and gains were maintained at 3- and 12-month follow-ups [40].

In addition to documenting treatment outcome, it is important from a clinical point of view to report information on the predictors of treatment outcome [41,42]. In CBT for anxiety disorders, demographic variables (i.e., age, sex and socioeconomic status) are usually not significant predictors of treatment outcome and treatment adherence [42,43,44,45,46,47]. Low motivation for treatment and poor working alliance, which are factors common to all psychotherapies, have often been associated with a poorer treatment response [42,48,49,50,51]. Change in intolerance of uncertainty, a putative process specific to CBT for GAD, has been shown to mediate treatment outcome and to precede changes in the symptoms of GAD [52]. Predictors of outcome should also be examined when CBT is delivered by VCP to help clinicians adapt their interventions. In their non-inferiority and non-randomized controlled trial, Bouchard et al. [34] and its online supplement found that motivation and working alliance were not statistically significant predictors of improvement for CBT for panic disorder delivered by VCP. As predicted by CBT models of panic disorder, change in dysfunctional beliefs about bodily sensations significantly predicted treatment outcome. No data are available on the predictors of treatment outcome when CBT is delivered by VCP for GAD, and more specifically, whether there is a difference in the role of intolerance of uncertainty when CBT-IU is delivered by VCP or face-to-face. 

The objectives of the current study are: (1) to assess with a randomized clinical trial the non-inferiority of CBT-IU for GAD delivered by VCP compared to face-to-face CBT-IU for GAD, and (2) to support findings from studies of face-to-face CBT-IU that show that changes in intolerance of uncertainty are associated with treatment outcome. Based on previous studies, we hypothesized that delivering CBT-IU by VCP would be statistically non-inferior to face-to-face therapy on primary, secondary and tertiary measures. The non-inferiority margin was defined a priori (in the grant application) by a strict and small margin of tolerance for non-inferiority of 10%, or a ε of 0.36 [53], p.16 which, for example, represents a difference in change between the two conditions of no more than 10% on the primary outcome measure. The primary outcome was the severity of GAD as assessed with a standardized structured diagnostic interview. The secondary outcomes were worry, intolerance of uncertainty and overall GAD symptoms. The tertiary outcomes, which focused on generalization of treatment outcome, were depressive mood and quality of life. For the second objective, it was hypothesized that change in intolerance of uncertainty would be significantly associated with treatment outcome, and that this relationship would not be due to shared variance with common therapy factors (motivation, working alliance, perceived therapist’s competence and client satisfaction) or the different treatment modalities (VCP or face-to-face therapy). The study was not designed to demonstrate the causal role of change in intolerance of uncertainty (as shown by Bomyea et al. [52]) but to document the relative contribution of specific and non-specific factors associated with the treatment outcomes of CBT-IU.

## 2. Materials and Methods

### 2.1. Participants

Participants were 148 adults of White ethnicity who met diagnostic criteria for GAD [1] (excessive anxiety and worry, difficulty to control the worry, anxiety and worry associated with three or more of the six somatic symptoms, significant clinical distress or impairment, and the disturbance not better explained by substance abuse, another medical condition or another mental disorder) as assessed with the diagnostic interview described in Section 2.4.1. Recruitment, which took place from March 2014 to December 2016, was conducted in university-based mental health clinics specialized in anxiety disorders across five of the six largest metropolitan areas in the Province of Quebec, Canada (in alphabetical order: Gatineau, Montréal, Québec, Sherbrooke and Trois-Rivières). Individuals responding to posters or articles in local newspapers, or referred by their doctors or mental health professionals were prescreened over the telephone (verbal consent was obtained prior to beginning the interview) and invited to attend an in-person diagnostic interview. Participants were eligible to take part to the study if they had a primary diagnosis of GAD, were aged between 18 and 75, and were fluent in French. Prior to study enrollment, participant also had to agree to abstain from starting, or refraining from changing, any antidepressant or anxiolytic medications and to not undergo any other psychotherapy during the course of treatment. Participants were excluded from the study if they received CBT in the previous 6 months or if they were taking any anxiolytic or antidepressant medication for less than, respectively, one or three months. Furthermore, individuals who received a secondary diagnosis of bipolar disorder, borderline personality disorder, intellectual disability, psychotic disorder, schizophrenia, substance-related disorder, or who presented firm suicidal intentions or a physical condition making participation in the study inadvisable (e.g., hearing impaired, visual impairment, epilepsy) were ineligible for participation. These eligibility criteria are similar to those used in recent efficacy trials of CBT for GAD [18]. 

**Figure 1:**
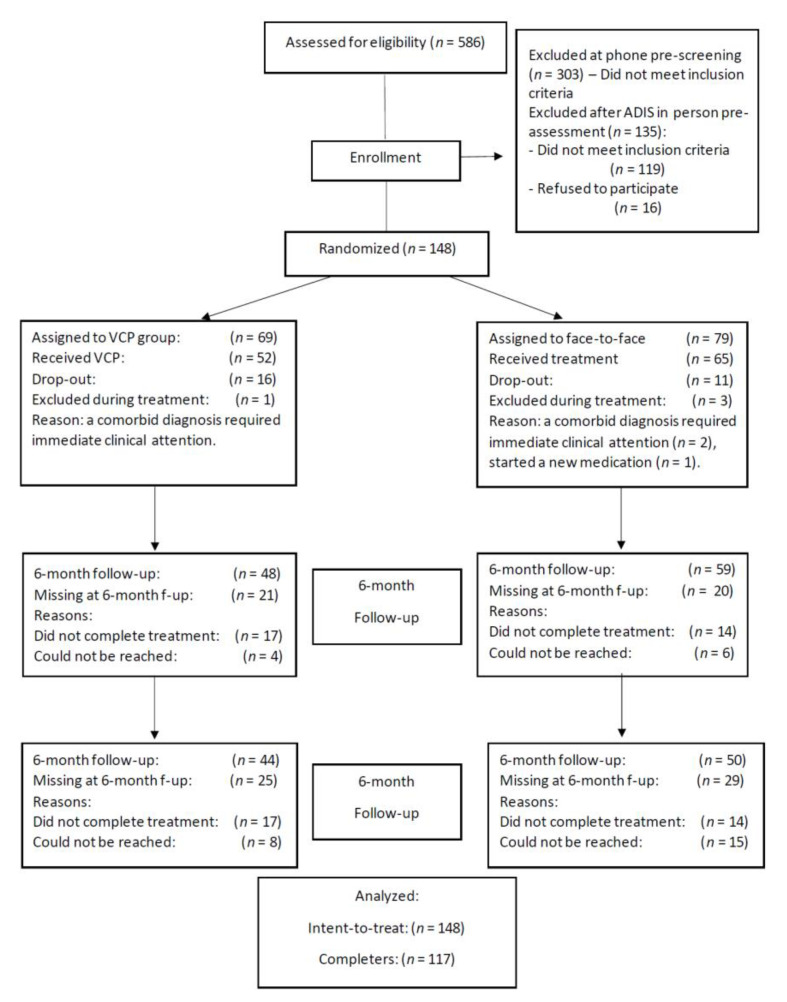
CONSORT flow chart of participation in the randomized control trial.

Figure 1 Details the flow of participants from recruitment to follow-up. Intent-to-treat analyses are reported in this article, with data for treatment completers reported in Appendix A. The sample size was established a priori based on a power analysis for non-inferiority trials [53,54].

### 2.2. Design and Ethics

This study received approval from the Research Ethics Board of the Université du Québec en Outaouais (Gatineau Campus, main research site) as well as from the Research Ethics Board of each participating center. Voluntary and informed consent, ethical reviews, and ethical approvals were performed in accordance with the Declaration of Helsinki of 1975, as revised in 2018, and the ethical standards of the Canadian Tri-Council 2018 policy statement for ethical conduct for research involving humans. Participants did not receive any monetary compensation for participating in this study. Because the cost of the treatment was covered by the research grant, receiving free treatment could be considered as an incentive. Yearly reports were submitted to the Research Ethics Boards, which monitored the study until termination (no adverse events were reported by participants). The study was designed, funded, and conducted in accordance with CONSORT (Consolidated Standards of Reporting Trials) guidelines for trials assessing non-pharmacological treatments and for non-equivalence trials [55,56]. The clinical trial was registered at the time the study received ethical approval and was expected to begin (ISRCTN12662027; https://doi.org/10.1186/ISRCTN12662027 (accessed on 30 August 2022), before participants were allocated to treatment conditions.

### 2.3. Recruitment and Randomization

All clinical interview sessions occurred in private offices on-campus. The interviews were administered by supervised graduate students in clinical psychology who had completed at least two years of practicum training. In order to assess inter-judge agreement on diagnosis, all clinical interviews were audio recorded. In the absence of a clear diagnostic consensus, a final diagnostic decision was reached after discussion between the interviewer and the senior researcher of the study site. Individuals who were deemed to be eligible to participate were informed of the implications of taking part in the study and signed the study’s comprehensive consent form. Recruitment was terminated in accordance with the timetable presented in the research grant to ensure feasibility of the 12-month follow-up. 

After providing informed consent, participants were randomized to one of the two treatment conditions: VCP or face-to-face psychotherapy. Randomization was generated before initiating recruitment and was performed using an iPhone generator of random numbers (i.e., 1, 2). A different randomization table was generated for each recruitment site. The study researchers were unaware of treatment allocation. If a participant refused to participate or dropped out of the study, their assignment slot was not reassigned to another participant. A second randomization was performed for participants assigned to the VCP condition in order to determine which distant VCP site would provide psychotherapy. The second randomization followed the same procedure as the first one, with the exception of the use of four randomizing options, numbered 1 to 4 (their local site was excluded from the second randomization). All participants received psychotherapy in an office at the local university-based mental health clinic where they had been recruited and received the diagnostic interview.

### 2.4. Assessment

All primary, secondary, and tertiary outcome measures were administered at pre-treatment, post-treatment, 6-month follow-up, and 12-month follow-up. All measures have been extensively validated and used in clinical trials. The length of the follow-up was established to provide data on long-term outcome. Follow-ups of 12 months are standard in CBT and feasible within funding constraints. The 6-month follow-up was planned to limit the risks of 12-month attrition and to limit the impact of carrying forward the last available observations for participants who were not able to attend the onsite 12-month diagnostic follow-up interview. Measures not specific to outcome or CBT-IU were: descriptive statistics and treatment motivation (assessed at pre-treatment), working alliance and perceived therapist competence (assessed at mid-treatment [57]). Note that analyses conducted with the measure of working alliance after the first, third and fifth therapy session yielded similar results. Treatment satisfaction was measured at post-treatment. To minimize social desirability, participants were informed that their psychotherapists would not have access to their responses concerning the working alliance and perceived therapist competence. Participants placed the questionnaires in an envelope and in a locked box accessible only to the study researchers. Ratings of treatment fidelity and statistical analyses were performed after the trial was completed. Data were entered at each site and sent to the main study site after the trial was completed for analyses.

#### 2.4.1. Primary Measure: Documenting Efficacy

Anxiety Disorders Interview Schedule for DSM-IV (ADIS-IV). The ADIS-IV [58] was initially used to establish the diagnosis of GAD and to identify other disorders (i.e., other anxiety disorders, mood disorders, obsessive-compulsive disorder, post-traumatic stress disorder, psychotic symptoms, somatization disorders, and substance use disorder). This semi-structured interview is commonly used as a treatment outcome measure in research on GAD. In addition to information about differential diagnosis, the ADIS-IV provides a score of clinical severity for each disorder, ranging from 0 (no symptom, distress or interference) to 8 (severe symptoms, distress or interference). A score of 4 and above warrants a diagnosis. Following the publication of the DSM-5 [1], the ADIS was revised for consistency with the DSM-5’s diagnostic criteria. However, the section on GAD was not revised, as its criteria remained unchanged from the DSM-IV to the DSM-5. The ADIS has demonstrated good inter-rater reliability for each anxiety disorder [58]. The ADIS was completed in person by an assessor blind to the treatment condition of the participant. Reassessment by blind raters using audio recordings of a subset of ADIS-IV interviews in this sample confirmed the excellent reliability of GAD scores (intraclass correlation = 0.94, *p* < 0.001).

#### 2.4.2. Secondary Measures: Documenting Self-Reported Symptoms of GAD and IU

Penn-State Worry Questionnaire (PSWQ). The PSWQ [59] is the most widely used measure of excessive worry, which is the central feature of GAD [20,60,61]. The questionnaire includes 16 items rated on a 5-point Likert scale ranging from 1 (not at all typical of me) to 5 (very typical of me). Eleven items are stated in the direction of pathological worry (e.g., « My worries overwhelm me »), while the remaining 5 items are inverted and reverse scored (e.g., « I find it easy to dismiss worrisome thoughts »). The PSWQ has strong internal consistency (Cronbach’s alpha of 0.95 [59]). Higher PSWQ scores indicate greater levels of worry. 

Worry and Anxiety Questionnaire (WAQ). The WAQ [62] is an 11-item self-report measure of the DSM-IV diagnostic criteria for GAD. Items are rated on a 5-point Likert scale ranging from 0 (not at all) to 8 (very severely), and they reflect both the cognitive and somatic symptoms of GAD. Three items assess cognitive symptoms (excessive or exaggerated worry, duration of excessive worry, difficulty controlling worry), six items assess somatic symptoms (restlessness, being easily fatigued, difficulty concentrating, irritability, muscle tension, sleep disturbance), and one item assesses interference. The WAQ total score is based on a weighted sum score ranging from 0 to 56, and higher WAQ scores indicate more severe GAD symptoms. It has been shown to possess strong internal consistency (Cronbach’s alpha of 0.90 [63]).

Intolerance of Uncertainty Scale (IUS). The IUS [64] is a self-report measure consisting of 27 items assessing negative beliefs about, and reactions to, uncertainty. It is a measure of the core variable underlying CBT-IU, not of GAD severity, and it has been shown by Bomyea et al. [52] to mediate treatment outcome. Items are rated on a 5-point scale from 1 (Not at all characteristic of me) to 5 (Entirely characteristic of me), with higher scores reflecting greater intolerance of uncertainty. The IUS has a high internal consistency (Cronbach’s alpha of 0.91). The total score is calculated by summing all items, with higher IUS scores indicating stronger intolerance of uncertainty.

#### 2.4.3. Tertiary Measures: Documenting Generalization to General Functioning

Beck Depression Inventory (BDI-II). The BDI-II [65] is one of the most widely used measures for assessing depressed mood. It includes 21 items in which four response options are presented on a 4-point Likert-type scale ranging from 0 to 3, with higher scores corresponding to higher levels of depressive symptoms. Respondents are asked to endorse statements regarding how they have been feeling over the past 2 weeks. The total score can vary from 0 to 63 and the measure has high internal consistency (Cronbach’s alpha of 0.90).

World Health Organization Quality of Life (WHO-QOL - Psychological and WHO-QOL - Social relations). The WHO-QOL brief [66] is a self-report questionnaire developed by the World Health Organization that aims to assess quality of life across different cultures. Quality of life is assessed by 6 items documenting psychological health and 6 items documenting social relationships. The WHO-QOL-Psychological scale (Cronbach’s alpha of 0.81) and the WHO-QOL-Social relations scale (Cronbach’s alpha of 0.71) document global personal and interpersonal functioning with adequate internal consistency; higher scores indicate a higher quality of life in these areas.

#### 2.4.4. General Measures Not Specific to Outcome and CBT-IU

Client Motivation for Therapy Scale (CMTS). Patients’ motivation to engage in therapy was assessed using the CMTS [67]. This scale, which is based on Self-Determination Theory [68], is made-up of 6 subscales: intrinsic motivation, integrated regulation, identified regulation, introjected regulation, external regulation and amotivation. The 24 items are scored on a 7-point Likert scale, ranging from 1 (not true at all) to 7 (totally true). The internal consistency of the scale is very good, with an average Cronbach’s alpha of 0.84. The total score was calculated as recommended by the authors [67], and a higher score expresses a stronger self-determined motivation to engage in psychotherapy.

Working Alliance Inventory (WAI). The WAI [69], patient-version, is the most widely used instrument for assessing the working alliance. Patients rate how they perceive their working alliance in terms of agreement on psychotherapy goals, psychotherapy tasks and emotional bond with their therapist. The complete patient version consists of 36 items rated on a 7-point Likert scale ranging from 1 (never) to 7 (always). This scale has an excellent Cronbach’s alpha of 0.93. Scores range from 36 to 252, and high scores indicate a strong working alliance. 

Therapist Competence Scale (TCS). The TCS [70] assesses the patient’s perception of their therapist’s competence during CBT. The total score on the original version is based on two subscales measuring technical skills and interpersonal skills with 20 items rated on a 7-point Likert scale ranging from 1 (strongly disagree) to 7 (strongly agree). It has been shown to differ from working alliance and patient motivation and to have a strong Cronbach’s alpha of 0.87. Higher scores indicate greater therapist competence according to patients.

Client Satisfaction Questionnaire (CSQ). The CSQ [71]. The CSQ is an 8-item self-report questionnaire assessing patient satisfaction with health services. Each item consists of a statement about the services received, and satisfaction is rated on a 4-point Likert scale. The total score ranges between 8 and 32, with higher scores indicating higher satisfaction. Cronbach’s alpha is strong at 0.92.

#### 2.4.5. Measures Used for Methodological Purposes

Structured Clinical Interview for DSM–IV Axis-II (SCID-II). The SCID-II [72] is a validated structured interview for assessing personality disorders. In this study, only the borderline personality disorder module was used. A 15-item self-administered questionnaire was first completed by participants to assess possible symptoms of borderline personality disorder, followed by the Borderline Personality Disorder semi-structured interview module of the SCID-II. People with a SCID-II borderline personality diagnosis were excluded from the study.

Adherence to the treatment manual. Psychotherapy sessions were video recorded to assess adherence to the treatment manual. A rating scale was used to document the extent psychotherapists respected the structure of the session (e.g., follow-up of home exercises, addressing intolerance of uncertainty, discussion, planning the upcoming home exercises) and whether each treatment strategy was addressed in the correct module. A quarter of the sessions of participants who completed the trial were randomly chosen, equally in each condition and within each module, to be examined by blind evaluators trained in CBT. Adherence to the treatment manual was calculated based on the frequency an element from the list was checked as being performed according to the manual, with a score of 100% corresponding to complete adherence to the manual. 

### 2.5. Treatment

Individuals allocated to face-to-face treatment were greeted at the local site by their assigned psychotherapist, whereas participants assigned to the VCP condition were met by a research assistant who set up the office with the videoconferencing system for VCP with the assigned clinician at the other site (remote site/another city). The research assistant used a remote control to call the remote site, adjust the volume and left the room just before the beginning of the VCP session. The assistant remained nearby and available in case of technical problems or emergencies. 

All patients received weekly manual-based CBT based on the IU model of GAD [73,74]. In the face-to-face condition, the patient and psychotherapist met physically in the same room. In the VCP condition, the patient and psychotherapist met online; the patient was in a room at the local site (i.e., the university-based clinic where the patient received the diagnostic interview) and the clinician was in a room in the remote site (i.e., a university-based clinic in another city, where the clinician delivered CBT-IU). 

The treatment protocol was identical for both conditions and consisted of 15 weekly, 60-min sessions divided into six modules: (Module 1, Session 1) building a working alliance, a shared case formulation and providing psychoeducation on the symptoms of GAD and the principles of CBT; (Module 2, Session 2) re-evaluating the usefulness of worrying; (Module 3, up to three sessions) increasing tolerance to uncertainty; (Module 4, up to four sessions) improving problem solving and problem orientation; (Module 5, up to four sessions) written exposure to worry; and (Module 6, Sessions 14 and 15) wrap-up and relapse prevention. The use of a treatment manual based on content that must be addressed by module (as opposed to content that must be addressed at each session) allowed for some clinical flexibility in adjusting the pace of treatment while maintaining the delivery of a reproductible validated intervention. Psychotherapists were required to proceed through all six modules and were not allowed to add new material or deviate from the planned sequence; however, they had the freedom to deviate from the expected pace by one or two sessions. A written treatment manual was provided to psychotherapists and to patients, with home exercises scheduled between sessions. Ratings of adherence to the treatment manual were 92.35% (SD = 9.45) in the VCP condition and 94.31% (SD = 8.33) in the face-to-face therapy condition. No significant difference in integrity was found between the two treatment conditions *(t*(103) = 1.12, bilateral *p* = 0.267, partial eta squared = 0.012) or the five sites (F(4,100) = 1.89, *p* = 0.117, partial eta-squared = 0.0710).

Psychotherapists were 23 graduate students (91% female) in clinical psychology trained in CBT, who received weekly supervision by the study researchers (six by SB, five by AM, five by GB, four by FL and three by PG, all registered psychologists trained in CBT-IU). One onsite meeting was conducted prior to starting the RCT to review the treatment protocol and ensure the homogeneity of treatment delivery among sites. One hour of the meeting was also devoted to train all staff members on how to use the VCP system. All psychotherapists provided CBT to participants in both conditions (i.e., they were not assigned to provide treatment in only one of the two conditions). 

### 2.6. Material

The technology used for this RCT required dedicated videoconference systems (i.e., not software used on a computer or a portable device), as this technology was the standard in communication security at the time the trial was conducted (standard H.323 with the use of a Gatekeeper). The hardware consisted of Tandberg MXP90 videoconference codec systems in each site allowing data transmission between sites at 1.544 Mbp/s through secured IP link, a 32-inch TV monitor, and a video camera located on the top of the TV monitor. In the VCP condition, the height of the TV monitor and the distance between monitor and patient’s chair were intended to replicate the distance and position of a psychotherapist when seated face-to-face. In the event of technical problems, a telephone and a list of telephone numbers of the other university-based clinics were available in all psychotherapy offices. If required, documents could be transmitted by email. The picture-in-picture option was activated on the psychotherapists’ videoconference system, and the psychotherapy sessions were recorded on a videorecorder to assess fidelity to the treatment protocol. When psychotherapy was delivered face-to-face, the local videoconference system was turned on (with the TV monitor turned off) to record psychotherapy sessions on the videorecorder.

## 3. Results

### 3.1. Statistical Analyses

Data were analyzed using IBM SPSS version 28 (IBM Corp, Armonk, NY, USA, 2021), except for non-inferiority tests which were conducted with jamovi 2.2.5 [75] and the TOSTER module [76] for the lower or upper bound 1-sided significance tests and probability values. Non-inferiority analyses were conducted in accordance with recommendations by Mauri and D’Agostino [77] and Wellek’s [53], (p. 16, p. 30) using a strict margin of tolerance for non-inferiority of ε = 0.36 (i.e., ±10%) applied to the lower equivalence bound of the TOST equivalence test when VCP showed smaller improvements in comparison to face-to-face or applied to the higher equivalence bound of the TOST equivalence test when VCP showed larger improvements in comparison to face-to-face. The non-inferiority margin was set a priori in the grant proposal based on clinical expertise and Wellek’s [53] strict criteria of smallest acceptable difference. Missing data were handled using an intent-to-treat approach, where each missing information was replaced by the last available observation carried forward. This approach is more conservative than analyzing only treatment completers, as it protects against inflation of success rates at post-treatment and follow-ups. The non-completers rate was not statistically different in the two conditions (X2(1) = 1.06, *p* = 0.30). Further comparisons between completers and non-completers revealed no statistical difference on most variables (age, sex, presence or number of comorbid disorders, treatment sites, ADIS-IV, WAQ, and IUS) but significantly lower scores among the non-completers in motivation, PSQW, and measures of quality of life, as well as higher BDI-II scores. Comparisons between completer status and treatment conditions revealed no statistically significant interaction, with all effect sizes being very small (partial eta square range between 0.000 and 0.01). Although the impact of non-completers seemed limited, following recommendations by Mauri and D’Agostino [77], results of the per-protocol treatment completers is also reported for consistency of the non-inferiority tests. As an alternative for handling missing data and covariance among measures over time, Mixed Linear Modeling (MLM) analyses are reported in Appendix A. Analyses on completers are reported in the main text to rely on actual data from participants instead of data estimated by MLM models. Repeated measures ANOVAs with completers and MLM analyses will be given less attention, as their conclusions were consistent with the more conservative approach. The assumption of normality was not met for the outcome measures, which is expected in clinical samples. All analyses were also performed with non-parametric analyses, and all results of the parametric analyses were replicated; parametric analyses are therefore reported here. Mauchly’s test for sphericity was sometimes statistically significant; hence, the Greenhouse–Geisser correction to the degrees of freedom was applied to all analyses. Significance levels were set at *p* = 0.05 for the descriptive statistics and the main outcome measure (ADIS-IV), and family-wise Bonferroni corrected for the secondary (PSWQ, WAQ, IUS: *p* < 0.05/3 = 0.017) and tertiary measures (BDI-II, WHO-QOL—Psychological, WHO-QOL—Social relations: *p* < 0.05/3 = 0.017). 

To address the second objective of the study, participants with missing data at Session 7 or at post-treatment were excluded from the regression analyses. Changes from pre to post-treatment for the multiple hierarchical regression involving the ADIS-IV and IUS were calculated using residualized change scores (results were similar to those obtained when pre and post scores were used in the regression, but with greater degrees of freedom and power). Predictors of pre/post residualized change on the ADIS-IV entered into the regression were: treatment condition, psychotherapist treatment site, motivation toward therapy at Session 1, working alliance at Session 7, perceived psychotherapist competence at Session 7, client satisfaction at post-treatment and pre/post residualized change in intolerance of uncertainty. The hierarchical regression tested the role of change in intolerance of uncertainty over and above variables that are non-specific to CBT for GAD. A treatment by change in IUS interaction parameter was further used to test for a between-condition difference in the role of cognitive change. The significance level was set at 0.05 for the regression analyses.

### 3.2. Description of the Sample

Table 1 presents descriptive information for the VCP and face-to-face conditions for the complete intent-to-treat sample. Chi-square tests and Student’s t-tests did not reveal pre-existing differences between the two conditions, except for working alliance and perceived therapist competence which reached statistical significance in a direction opposite to the non-inferiority hypotheses (i.e., suggested superiority of VCP over face-to-face therapy) and did not require additional statistical corrections for the non-inferiority tests. There was no statistically significant difference when comparing recruitment sites (i.e., the five centers, with participants assigned to VCP receiving treatment by psychotherapists from other centers) and psychotherapist treatment sites (i.e., the site of the psychotherapists, regardless of treatment modality) on all variables at pre-treatment.

### 3.3. Main Outcome and Non-Inferiority Analyses

Descriptive statistics at each time point are reported in Table 2, and results of the non-inferiority analyses are reported in Table 3. The repeated measures ANOVAs confirmed statistically significant and large time effects for each variable. Contrasts comparing pre and post-treatment were all statistically significant, and effect sizes were large for the ADIS-IV (pre/post F(1,146) = 211.08, *p* < 0.001, partial eta-squared = 0.59; pre/6-month follow-up F(1,146) = 192.6, *p* < 0.001, partial eta-squared = 0.57; pre/12-month follow-up (1,146) = 191.32, *p* < 0.001, partial eta-squared = 0.57), the PSWQ (pre/post F(1,146) = 177.78, *p* < 0.001, partial eta-squared = 0.55; pre/6-month follow-up F(1,146) = 202.19, *p* < 0.001, partial eta-squared = 0.58; pre/12-month follow-up (1,146) = 191.29, *p* < 0.001, partial eta-squared = 0.57), the WAQ (pre/post F(1,146) = 167.9, *p* < 0.001, partial eta-squared = 0.54; pre/6-month follow-up F(1,146) = 204.08, *p* < 0.001, partial eta-squared = 0.58; pre/12-month follow-up (1,146) = 187.93, *p* < 0.001, partial eta-squared = 0.56), the IUS (pre/post F(1,146) = 143.21, *p* < 0.001, partial eta-squared = 0.50; pre/6-month follow-up F(1,146) = 165.4, *p* < 0.001, partial eta-squared = 0.53; pre/12-month follow-up (1,146) = 168.83, *p* < 0.001, partial eta-squared = 0.54], the BDI-II (pre/post F(1,146) = 95.14, *p* < 0.001, partial eta-squared = 0.40; pre/6-month follow-up F(1,146) = 108.75, *p* < 0.001, partial eta-squared = 0.43; pre/12-month follow-up (1,146) = 92.97, *p* < 0.001, partial eta-squared = 0.39), the WHO-QOL-Psychological subscale (pre/post F(1,146) = 60.58, *p* < 0.001, partial eta-squared = 0.31; pre/6-month follow-up F(1,146) = 69.42, *p* < 0.001, partial eta-squared = 0.34; pre/12-month follow-up (1,146) = 71.25, *p* < 0.001, partial eta-squared = 0.34], and the WHO-QOL-Social relations subscale (pre/post F(1,146) = 16.42, *p* < 0.001, partial eta-squared = 0.11; pre/6-month follow-up F(1,146) = 30.51, *p* < 0.001, partial eta-squared = 0.18; pre/12-month follow-up (1,146) = 30.6, *p* < 0.001, partial eta-squared = 0.18).

Main effects for all conditions were statistically non-significant (see Table 3), with partial eta-squares of 0.001 for the ADIS-IV, 0.01 for the PSQW, 0.006 for the WAQ, 0.009 for the IUS, 0.000 for the BDI-II, 0.004 for the WHO-QOL-Psychological subscale, and 0.009 for the WHO-QOL-Social relations subscale, respectively.

Time by Condition interaction contrasts comparing CBT-IU delivered by VCP and delivered face-to-face revealed very small effect sizes for all variables: ADIS-IV (pre/post F(1,146) = 0.006, *p* = 0.938, partial eta-squared = 0.000, difference in improvement = −0.03; pre/6-month follow-up F(1,146) = 0.762, *p* = 0.384, partial eta-squared = 0.005, difference in improvement = −0.29; pre/12-month follow-up (1,146) = 0.667, *p* = 0.42, partial eta-squared = 0.005, difference in improvement = −0.27), the PSWQ (pre/post F(1,146) = 0.822, *p* = 0.323, partial eta-squared = 0.007, difference in improvement = 2.1; pre/6-month follow-up F(1,146) = 1.364, *p* = 0.245, partial eta-squared = 0.009, difference in improvement = 2.6; pre/12-month follow-up (1,146) = 1.314, *p* = 0.245, partial eta-squared = 0.009, difference in improvement = 2.5), the WAQ (pre/post F(1,146) = 0.024, *p* = 0.877, partial eta-squared = 0.000, difference in improvement = −0.3; pre/6-month follow-up F(1,146) = 0.159, *p* = 0.69, partial eta-squared = 0.001, difference in improvement = 0.8; pre/12-month follow-up (1,146) = 0.1.907, *p* = 0.169, partial eta-squared = 0.013, difference in improvement = 2.8), the IUS (pre/post F(1,146) = 0.138, *p* = 0.711, partial eta-squared = 0.001, difference in improvement = 1.4; pre/6-month follow-up F(1,146) = 0.593, *p* = 0.442, partial eta-squared = 0.004, difference in improvement = 2.9; pre/12-month follow-up (1,146) = 0.311, *p* = 0.578, partial eta-squared = 0.002, difference in improvement = 2.1), the BDI-II (pre/post F(1,146) = 0.257, *p* = 0.613, partial eta-squared = 0.000, difference in improvement = 0.88; pre/6-month follow-up F(1,146) = 0.006, *p* = 0.938, partial eta-squared = 0.000, difference in improvement = −0.13; pre/12-month follow-up (1,146) = 0.496, *p* = 0.482, partial eta-squared = 0.003, difference in improvement = 1.26), the WHO-QOL-Psychological subscale (pre/post F(1,146) = 0.213, *p* = 0.645, partial eta-squared = 0.002, difference in improvement = 0.14; pre/6-month follow-up F(1,146) = 0.387, *p* = 0.535, partial eta-squared = 0.003, difference in improvement = −0.26; pre/12-month follow-up (1,146) = 0.720, *p* = 0.398, partial eta-squared = 0.005, difference in improvement = 0.37), and the WHO-QOL-Social relations subscale (pre/post F(1,146) = 0.179, *p* = 0.673, partial eta-squared = 0.001, difference in improvement = 0.16; pre/6-month follow-up F(1,146) = 0.347, *p* = 0.557, partial eta-squared = 0.003, difference in improvement = 0.52; pre/12-month follow-up (1,146) = 0.198, *p* = 0.657, partial eta-squared = 0.001, difference in improvement = 0.35). 

**Figure 2:**
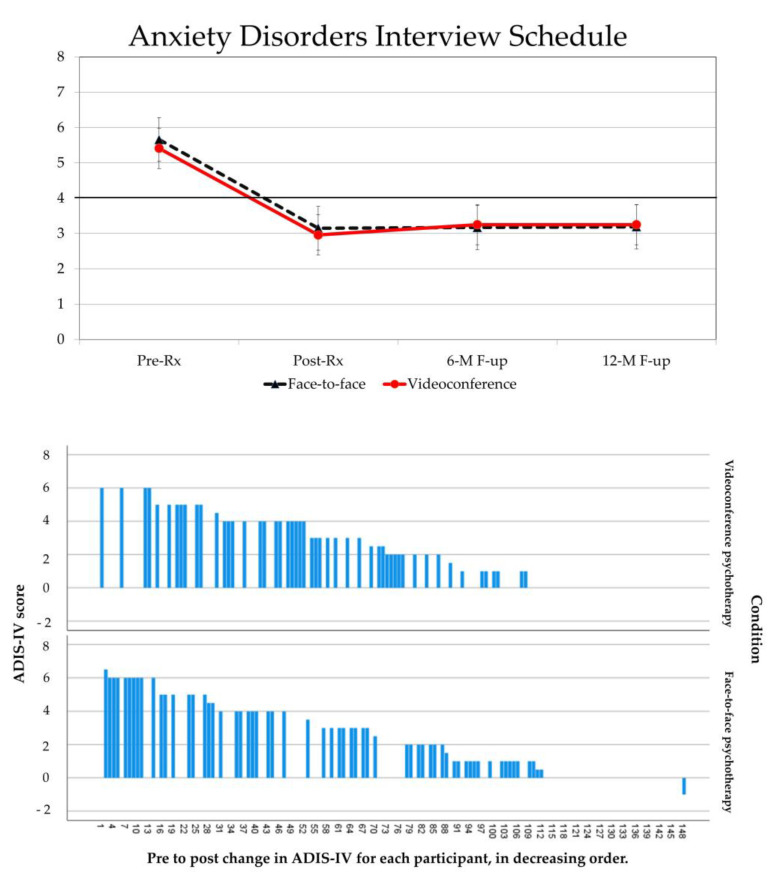
Visual representations of the results on the ADIS-IV primary outcome measure for 148 adults diagnosed with GAD who received cognitive-behavior therapy in videoconference (VCP) or face-to-face, with data aggregated by condition (line-graph, top) and pre to post change data reported for each individual (waterfall bar chart, bottom).

The non-inferiority tests (Table 3) showed that the impact of the treatment was statistically non-inferior when delivered by VCP compared to face-to-face for all measures. For the primary measure of efficacy (ADIS-IV), mean scores improved from pre to post-treatment on average by 44.47% in the VCP condition and by 42.44% in the face-to-face condition. Using the cut-off severity score of 4 on ADIS-IV, 68% participants in the VCP condition no longer met diagnostic criteria for GAD (vs. 57% in the face-to-face condition) at post-treatment, 64% (vs. 61%) at 6-month follow-up, and 65% (vs. 62%) at 12-month follow-up. No statistical analyses were conducted on remission rates to avoid redundancy with the ADIS-IV severity scores. A visual representation of the results is provided in Figure 2 in the form of a line chart with 95% confidence intervals and a waterfall bar chart of each individual’s change from pre to post-treatment. Only five participants who completed the treatment (one in VCP, four in face-to-face therapy) reported no improvement on the ADIS-IV at post-treatment. The remaining participants with no change illustrate the impact of the intent-to-treat methodology. One participant (in the face-to-face condition) reported a deterioration of 1 point at post-treatment. 

As recommended [77], non-inferiority analyses were also conducted with treatment completers (i.e., per protocol; see also Appendix A for results from MLM analyses). The analyses of the treatment completers sample replicated the results from the intent-to-treat sample, as shown in Table 4, with the exception of the measure of change in global psychological quality of life from pre-treatment to 6-month follow-up. In this case, the probability of the non-inferiority tests was lower than 0.05 (*p* = 0.03) but did not reach the Bonferroni corrected level of significance due to lack of power (see the Figure in Appendix A for a visual illustration of the results). If the tolerance margin for defining a difference as negligible would have been set at 12% instead of 10%, the non-inferiority test would have met the Bonferroni correction. Using the cut-off severity score of 4 on ADIS-IV, 90% participants in the VCP condition no longer met diagnostic criteria for GAD (vs. 67% in the face-to-face condition) at post-treatment, 85% (vs. 72%) at 6-month follow-up, and 87% (vs. 74%) at 12-month follow-up.

To document the potential effects of sex, the presence of at least one comorbid disorder, medication use, previous psychotherapy, recruitment site, and psychotherapist site, analyses were conducted by considering these variables as independent factors and repeating the main repeated measures analyses for each factor. The only statistically significant interaction was found for the effect of psychotherapist site for the measures of depressed mood and quality of life in social relations. The statistically significant interaction effects for psychotherapist site did not influence the results of the non-inferiority analyses (i.e., what was statistically significant remained statistically significant, and vice versa), but the findings needed to be investigated. The Time by Treatment condition by Psychotherapist site interaction was statistically significant for the BDI-II (F(8.24,414) = 2.1, *p* = 0.034, partial eta-squared = 0.06) and the WHO-QOL-Social relations subscale (F(9.66,384) = 1.93, *p* = 0.042, partial eta-squared = 0.06). Probing the interactions revealed that the Time by Treatment condition interaction contrast was statistically significant for two psychotherapist sites. Face-to-face CBT seemed more impactful on these two measures than VCP when comparing the effect of psychotherapists from the Montréal site to those from the Gatineau site. A detailed exploration of the data revealed that there was less comorbidity in participants treated face-to-face by psychotherapists from the Montréal site (*n* = 16 co-diagnosed disorders) than from the Gatineau site (*n* = 26 co-diagnosed disorders). The difference in number of comorbid diagnoses per participant was not statistically significant (F(1,67) = 0.41, *p* = 0.53, partial eta-squared = 0.006), but the number of comorbid disorders was statistically significantly associated with more severe depressed mood (*r =* 0.37, *p* = 0.002) and lower quality of life in social relations (*r =* −0.28, *p* = 0.027) at pre-treatment. 

### 3.4. Predictors Change in GAD Severity at Posttreatment

Factors potentially associated with treatment efficacy, as measured by the residualized change in ADIS-IV scores from pre to post-treatment, were examined in a hierarchical regression analysis. The common therapy factors (i.e., motivation, working alliance, perceived therapist competence and client satisfaction) were entered in the first step of the regression. Treatment condition (VCP or face-to-face therapy) and psychotherapist treatment site were also entered as methodological controls. The factor specific to CBT-IU, change in intolerance of uncertainty, was entered in the second step to test its contribution to the regression model over and above the factors entered in the first step. The final regression model was statistically significant (F(7,113) = 7.33, *p* < 0.001, *R^2^* = 0.32, Adjusted *R*^2^ = 0.27). The second step in the hierarchical regression significantly contributed to the final model (F change (1,113) = 41.48, *p* < 0.001, change in *R^2^* = 0.25). Table 5 details the contribution of each variable to the final model. Consistent with the non-inferiority finding for the Time by Condition interaction with the IUS, testing the direct impact of treatment conditions on the residualized change in intolerance of uncertainty was not statistically significant (F change (1,112) = 0.031, *p* = 0.86, change in *R^2^* = 0.00) and did not reduce the significant role of intolerance of uncertainty in the regression (*t* = 6.99, *p* < 0.001, semi-partial correlation = 0.52). To support the discussion of the findings, Table 6 shows the correlation among the various measures used in the regression. In the hierarchical regression, the role of sex, age, income, education, living alone, medication use, previous psychotherapy and the presence of at least one comorbid disorder were also explored. None of the aforementioned variables significantly predicted outcome or changed the conclusions of the regression analysis.

## 4. Discussion

The motivation for the current study was to show that delivering CBT for GAD by videoconference would not be detrimental or different from delivering it face-to-face. The study was built on a non-inferiority randomized controlled design, with the intent to show VCP’s effectiveness at post-treatment and over the long-term using a small margin of tolerance in difference in outcome. Based on previous findings from studies of face-to-face psychotherapy, it was also hypothesized that change in intolerance of uncertainty would make a statistically significant contribution to the prediction of treatment improvement over and above the effect of non-specific predictors of outcome such as motivation, working alliance, perceived therapist competence, and treatment satisfaction.

The sample consisted of adults from five different sites, with clinically severe GAD, significant depressed mood, and high comorbidity rates. CBT-IU led to statistically significant and very large changes on all measures, with statistically significant non-inferiority results based on a margin of tolerance of 10%. The analyses confirmed, with an RCT conducted according to CONSORT guidelines, what has been found in previous clinical trials for other anxiety disorders [30,32,34,77] and other non-anxiety disorders [26,28,33]. In the current study, empirical evidence was obtained for the treatment of GAD. Specifically, treatment efficacy, as measured with severity ratings from the ADIS-IV, was large and maintained at follow-ups. None of the data on the primary measure of efficacy suggested that VCP may be less effective than face-to-face psychotherapy. When the impact of the treatment was compared using two other measures of GAD severity, the PSWQ and the WAQ, the non-inferiority findings remained the same, even after correcting for the number of comparisons. General measures of improvement such a depressed mood and quality of life also revealed a lack of significant difference within the strict margin of tolerance set a priori. The analyses were performed on the intent-to-treat sample and were essentially replicated using the treatment completers sample. Although the results of the current study were somewhat expected given previous findings [16,17,18,19,20,21], the efficacy of CBT-IU had yet to be conclusively documented with VCP. 

Documenting the predictors of change in the severity of GAD was important. The finding that change in intolerance of uncertainty was statistically non-inferior in VCP compared to face-to-face therapy, and significantly associated with treatment success, is consistent with expectations about CBT for GAD [12,52]. In the hierarchical multiple regression predicting change in ADIS-IV scores, change in intolerance of uncertainty was the only statistically significant predictor. The statistically limited role of working alliance, motivation and psychotherapist competence in predicting treatment outcome has sometimes been reported in studies on CBT [34,78] and must not be interpreted negatively. In the current study, these variables were significantly related to client satisfaction and, most importantly in the case of the working alliance, to change in intolerance of uncertainty. Their role must be interpreted in the context of comparing non-specific versus specific predictors of improvement in a manualized treatment for an anxiety disorder. In CBT, working alliance, motivation, competence and satisfaction are considered prerequisites for effective psychotherapy [79]; they enable patients to engage in changing the dysfunctional patterns that maintain their disorder. They are expected to be statistically related to treatment outcome. However, predictors of change based on validated psychopathology models of specific mental disorders should be more important predictors of outcome than non-specific predictors when it comes to treatments based on these models. Until now, that remained to be established with VCP for CBT-IU of GAD. Other forms of psychotherapy and other mental disorders may require a different investment to build, nurture and negotiate ruptures in alliance [57]. In terms of the dynamic mechanisms of CBT, Bouchard et al. [80] analyzed the role of working alliance in VCP and showed that telepresence (the feeling of being in the same psychotherapy room as the therapist) facilitates the development of a sound working alliance, which in turn enables patients and therapists to work on changing the dysfunctional behaviors and mental associations with perceived threat that are required for the reduction in the symptoms of anxiety disorders. 

Differential statistical analyses for sex, the presence of a comorbid disorder, taking medication, previous psychotherapy, recruitment site, and psychotherapist site did not reveal any statistically significant difference in VCP versus face-to-face therapy, except for the impact of psychotherapist site on measures of depressed mood and quality of life in interpersonal relations. The relative impact of VCP compared to face-to-face therapy differed on these two variables when comparing two specific sites. These unexpected differences did not influence the conclusions of the main analyses and were observed only on variables measuring the generalization of results. The treatment was manualized, adherence to the manual was excellent, there was no significant psychotherapist site difference in perceived therapist competence, severity of depressed mood, quality of life, treatment outcome on all other variables, or the presence or absence of a comorbid disorder. A potential explanation is that psychotherapists in one center had to deal with slightly less comorbid cases randomly assigned to face-to-face therapy than other centers and conditions. However, the presence of comorbid disorders was not associated with treatment outcome. The impact of comorbidity on how VCP generalizes to factors not specifically targeted in the treatment manual during CBT warrants further investigation. 

Contrary to expectations, participants in the VCP condition reported working alliance and perceived psychotherapist competence scores at Session 7 that were significantly higher than those of participants in the face-to-face condition. The statistically significant difference in the strength of the working alliance was not observed in this sample after the first few therapy sessions [81], and it is therefore not likely to be attributable to the effect of randomization. Interestingly, working alliance was significantly and strongly correlated with perceived therapist competence and treatment satisfaction, and to a smaller degree with change in intolerance of uncertainty. Perceived therapist competence was also significantly correlated with treatment satisfaction. In the context of a non-inferiority trial, these results were interpreted as suggesting that VCP may not be less effective than face-to-face CBT (for more on the treatment alliance in VCP, see also [34,82,83,84]). It is possible that in the current study, which was conducted before the COVID-19 pandemic, patients perceived their psychotherapists as more competent in the VCP condition, because of the additional challenge imposed by the use of technology, which positively impacted their working alliance. Further studies are required to explain why, under some circumstances, working alliance and perceived psychotherapist competence could be stronger after a few sessions in VCP than in face-to-face psychotherapy.

The CBT protocol used in this study focused on intolerance of uncertainty, was based on the 6-module version of CBT-IU, used cognitive restructuring and exposure strategies, and lasted 15 weeks. Can these findings be generalized to variations in CBT for GAD, for example a treatment based on a different theoretical model, e.g., acceptance-based behavior therapy [85], without a problem-solving module, using mostly behavioral experiments [18], or shorter in duration? Empirical findings and replications are always important [86], but the key question is why these variations would be influenced by delivering CBT by videoconference. For psychotherapeutic modalities that may be less compatible with VCP (e.g., group psychotherapy [87]), or for those that place an additional strain on motivation or working alliance (e.g., more intensive treatments), it is imperative to test their effectiveness when using videoconference. For more “standard” treatments, non-inferiority is very likely to be replicated. However, conducting clinical trials to document the efficacy, or non-inferiority, of VCP for other disorders than anxiety-related disorders and major depression remains pressing. Indeed, most studies using videoconferencing have been conducted on anxiety-related disorders and major depression [26,29,30,32,33,88]. The COVID-19 pandemic revealed the relevance of VCP and the lack of information about its efficacy for several disorders. Although not all mental disorders and forms of psychotherapy have solid empirical support when the treatment is delivered face-to-face, there is a need for many more clinical trials using VCP and other eHealth modalities [27]. 

Research on VCP has significantly evolved from the early clinical trials decades ago [89,90,91]. Until recently, it was not possible to recommend using the Internet to conduct VCP with sufficient levels of quality and confidentiality. The leap in technology over the last few years, between the moment the current study began and finished, is impressive. It is now possible for patients to receive VCP without having to commute to specific locations where secured and expensive videoconference systems are installed on dedicated communication networks, as was the case for the current study. This turn of events raises questions about the generalizability of our results and suggests several new lines of research. In the current study, the physical settings for the psychotherapy sessions were those one would expect for a mental health clinic. Now, patients can engage in VCP sessions at home, at work between meetings, or in a public café using the free unprotected public Wi-Fi network with a smartphone while simultaneously consulting other information on a laptop. Experimental studies are beginning to examine how to set up VCP to replicate face-to-face communication using computer-based systems (such as replicating direct eye contact [92]) and how these differences in settings can impact the psychotherapeutic process. For example, Grondin et al. [93] reported that altered eye contact in VCP may not be as detrimental as previously believed for the perception of empathy from a psychotherapist. Several practical questions remain unanswered, and these could have an impact on the contexts in which our results could be generalized, such as how to ensure that patients are in an adequate environment for psychotherapy (i.e., no distracting stimuli, secured confidentiality), which software options really make a significant contribution to an optimal experience (e.g., using the picture-in-picture option [94]), or which factors contribute to patients being ready to start their psychotherapy session (e.g., regaining composure in a waiting room before a session [95]). The past experiences of patients and psychotherapists using videoconference also merit study, as attraction to novelty and previous negative experiences with the technology may influence the impact of VCP [96]. Documenting how VCP may reduce barriers for accessing care also deserves to be studied more thoroughly [24]. 

Additional limitations of this study must be acknowledged. The study was conducted with volunteers of White ethnicity who agreed to be randomized either to face-to-face CBT or CBT by VCP. Results may not fully apply to people from other cultures or who do not have the choice of receiving VCP or not, either because of public health safety recommendations (e.g., imposed confinement due to COVID-19) or lack of a better alternative. In addition, participants were all motivated to engage in psychotherapy, which may not always be the case in routine clinical practice. The CBT-IU treatment protocol [74] used has evolved over the years to focus more specifically on intolerance of uncertainty [18], and treatment strategies such as problem-solving are no longer germane to the protocol. The treatment was delivered by graduate students in clinical psychology, which is a population that has been shown to be able to successfully use VCP to deliver evidence-based treatment [97]. More seasoned psychotherapists may hesitate to use VCP for a variety of reasons [96,98], may diverge from the application of already established treatment manuals to adapt the treatment to their liking [99], or may use exposure strategies less frequently than recommended [100]. The working alliance was measured with the total score, as opposed to focusing on each subscale [101], and according to patients’ perspective. Analyses of the psychotherapists’ perspective confirmed the quality of the working alliance in our sample [81], but analyses of video recordings of the therapy sessions would reveal much more interesting and nuanced observations of the working alliance, of how ruptures in alliance are handled and of how ruptures in acceptance of VCP technology could impact intersubjectivity and the treatment [102].

## 5. Conclusions

This study confirmed that CBT for GAD can be effectively delivered using videoconference technology. The nature of the treatment and it processes do not seem to be significantly disrupted when delivered remotely. Studies on VCP pave the way for a paradigm shift with regard to access to psychotherapy. Access to psychotherapy has traditionally been limited to geographical proximity. Patients can now access the psychotherapist of their choice despite geographic limitations. Mental health professionals can practice psychotherapy from their preferred location, including from rural areas with patients in urban centers. Access to a broader set of mental health professionals could help overcome cultural and expertise barriers faced by patients. However, this new situation has created challenges for organizations and governments providing mental health services. Patients can now ask for services from organizations that are not in their catchment area. Professionals can offer services based on their specialized expertise to patients in catchment areas that differ from the one served by their organization. The demands for services that cross legislative barriers will expand. Consequently, researchers will be called upon to provide empirical evidence to guide decisions about for whom VCP is appropriate, when and under which circumstances.

## Figures and Tables

**Table 1:** Descriptive statistics of the intent-to-treat sample of participants diagnosed with generalized anxiety disorder who received cognitive-behavior therapy either by videoconference (VCP) or face-to-face (FF).

	VCP (*n* = 69)	FF (*n* = 79)	Statistical Test (Chi-Square or *t* Test)
Age, mean (SD)	41.35 (14.80)	39.38 (16.23)	−0.77, *p* > 0.05
Sex (female)	57 (82.60%)	65 (82.30%)	0.003, *p* > 0.05
Presence of at least one comorbid disorder *	36 (52.2%)	44 (55.7%)	0.184, *p* > 0.05
Living alone	15 (21.70%)	8 (10.10%)	3.784, *p* > 0.05
Education High school	12 (17.40%)	13 (16.50%)	0.235, *p* > 0.05
College	21 (30.40%)	27 (34.20%)	
University	36 (52.20%)	39 (49.40%)	
Work status Full-time (35 h or +)	23 (33.30%)	26 (32.90%)	3.694, *p* > 0.05
Part-time (less than 35 h)	23 (33.30%)	23 (29.10%)	
Retirement	9 (13.00%)	11 (13.90%)	
Unemployment	11 (15.90%)	9 (11.4%)	
Other	3 (4.30%)	10 (12.70%)	
Annual income Lower than 29,999$	14 (20.90%)	27 (35.10%)	4.628, *p* > 0.05
(3 refused to answer) 30 k–59,999$	25 (37.30%)	19 (29.70%)	
60 k–89,999$	11 (16.40%)	14 (18.20%)	
90 k and more	17 (25.40%)	17 (22.10%)	
Taking medication	32 (46.4%)	25 (31.6%)	3.38, *p* > 0.05
Previous psychotherapy	49 (71.00%)	57 (72.20%)	0.23, *p* > 0.05
Motivation toward therapy (Session 1)	12.38 (4.60)	12.70 (3.67)	0.46, *p* > 0.05
Working alliance (Session 7)	233.18 (18.05)	225.80 (17.52)	−2.36, *p* < 0.05
Perception of therapist competence (Session 7)	164.00 (12.99)	156.47 (19.32)	−2.49, *p* < 0.05
Client Satisfaction (post-treatment)	28.32 (3.78)	27.77 (3.46)	−0.92, *p* > 0.05

Note. VCP = Videoconference psychotherapy; FF = Face-to-face psychotherapy; SD = Standard deviation. * Participants reported having up to four comorbid conditions and the number specific comorbid conditions were as follows: social anxiety disorder (*n* = 34), panic disorder (*n* = 20), agoraphobia (*n* = 14), major depressive disorder (*n* = 14), specific phobia (*n* = 14), obsessive-compulsive disorder or trichotillomania (*n* = 7), posttraumatic stress disorder (*n* = 4), other mood disorders (*n* = 7), eating disorder (*n* = 1), other (*n* = 6).

**Table 2:** Descriptive statistics for variables used in the non-inferiority analyses (intent-to-treat) (*n* = 148).

Variable	Condition	Pre		Post		6-Month F-Up		12-Month F-Up	
		M	SD	M	SD	M	SD	M	SD
ADIS	VCP	5.41	1.07	2.96	1.90	3.25	1.78	3.25	1.78
	FF	5.62	0.90	3.15	1.95	3.17	1.90	3.19	1.85
PSWQ	VCP	66.59	7.27	51.51	11.99	49.27	12.50	49.86	12.37
	FF	66.62	7.31	53.62	11.93	51.92	13.00	52.44	12.73
WAQ	VCP	42.85	6.46	29.87	12.84	28.09	12.71	27.54	12.59
	FF	43.50	6.25	30.20	12.96	29.54	12.06	30.99	11.62
IUS	VCP	85.13	20.54	61.96	23.71	59.51	22.87	58.68	23.20
	FF	87.07	19.33	65.30	22.24	64.34	23.38	62.80	22.58
BDI-II	VCP	21.52	10.93	12.61	11.03	12.68	10.22	12.29	11.03
	FF	21.16	8.96	13.13	11.67	12.19	11.37	13.19	11.03
QOL-Psychol	VCP	11.07	2.23	12.50	2.46	12.65	2.90	12.94	2.95
	FF	10.82	2.20	12.09	2.52	12.66	3.03	12.36	2.87
QOL-Social	VCP	12.22	3.16	13.17	3.44	13.90	3.64	13.80	3.85
	FF	11.84	2.87	12.61	3.11	13.20	3.39	13.19	3.10

Note. M = Mean; SD = Standard deviation; VCP = Videoconference Psychotherapy; FF = Face-to-face; ADIS = Anxiety Disorders Interview Schedule for DSM-IV, PSWQ = Penn-State Worry Questionnaire, WAQ = Worry and Anxiety Questionnaire, IUS = Intolerance of Uncertainty Scale, BDI-II = Beck Depression Inventory-II, QOL-Psychol = WHO-QOL-Psychological subscale, QOL-Social = WHO-QOL-Social relations subscale.

**Table 3:** Non-inferiority analyses of a RCT comparing the delivery of psychotherapy by videoconference or face-to-face to patients with generalized anxiety disorder (intent-to-treat) (*n* = 148).

Variable	Outcome Analysis-ANOVA				Non-Inferiority Analysis of the Statistical Interactions with a Strict Margin of Tolerance		
	Time F	Condition F	Interaction		Pre/Post	Pre/6-Month F-up	Pre/12-Month F-up
			F	eta squ.	W	W	W
ADIS	142.772 ***	0.095	0.617	0.004	2.27 **	3.07 **	3.01 **
PSWQ	151.523 ***	1.445	0.995	0.007	−3.16 ***	−3.35 ***	−3.32 ***
WAQ	129.289 ***	0.932	1.325	0.009	2.33 **	−2.57 **	−3.54 ***
IUS	129.984 ***	1.253	0.344	0.002	−2.54 **	−2.94 **	−2.72 **
BDI-II	72.406 ***	0.009	0.440	0.003	−2.66 **	2.25 **	−2.86 **
QOL-Psychol	45.621 ***	0.594	1.113	0.008	2.54 **	−2.75 **	3.03 **
QOL-Social	19.488 ***	1.293	0.182	0.001	2.49 **	2.71 **	2.77 **

Note. ADIS = Anxiety Disorders Interview Schedule for DSM-IV, PSWQ = Penn-State Worry Questionnaire, WAQ = Worry and Anxiety Questionnaire, IUS = Intolerance of Uncertainty Scale, BDI-II = Beck Depression Inventory-II, QOL-Psychol = WHO-QOL-Psychological subscale, QOL-Social = WHO-QOL-Social relations subscale. ANOVA results for the repeated pre–post by condition contrasts are reported in the text. W = Welch’s t-test. The value of the W is negative when the test is applied to the lower bound and positive when it is applied to the upper bound. ** *p* < 0.017, *** *p* < 0.001. eta squ = partial eta-squared (a measure of effect size of the differential impact of the treatment from one condition to the other).

**Table 4:** Non-inferiority analyses for participants who completed the treatment (*n* = 117).

Variable	Non-Inferiority Analysis of the Statistical Interactions with a Strict Margin of Tolerance		
	Pre/Post W	Pre/6-Month F-up W	Pre/12-Month F-up W
Anxiety Disorders Interview Schedule for DSM-IV	−2.98 **	2.01 *	−1.96 *
Penn-State Worry Questionnaire	−1.87 ***	−4.27 ***	−4.15 ***
Worry and Anxiety Questionnaire	−2.6 **	−3.38 ***	−4.56 ***
Intolerance of Uncertainty Scale	−3.06 **	−3.63 ***	−3.36 ***
Beck Depression Inventory-II	−2.91 **	−2.33 **	−3.12 **
WHO-QOL-Psychological subscale	2.94 **	−1.91 *	3.47 ***
WHO-QOL-Social relations subscale	2.52 **	2.81 **	2.86 **

Note. W = Welch’s t-test. The value of the W is negative when the test is applied to the lower bound and positive when it is applied to the upper bound. * *p* < 0.5, ** *p* < 0.017, *** *p* < 0.001.

**Table 5:** Contribution of non-specific and specific factors of CBT for GAD when delivered by videoconference or face-to-face at the second step of a hierarchical regression predicting improvements in ADIS-IV ratings.

	*std Beta*	*t*	*sig. p*	*Simple corr.*	*Partial corr.*	*Semi-Partial corr.*
Treatment condition	−0.12	−1.6	0.118	−0.11	−0.15	−0.12
Center providing psychotherapy	−0.03	−0.45	0.657	0.04	−0.04	−0.03
Motivation (Session 1)	−0.05	−0.67	0.502	−0.07	−0.06	−0.05
Working alliance (Session 7)	0.21	1.71	0.087	−0.07	0.16	0.13
Perceived therapists’ competence (Session 7)	−0.06	−0.65	0.52	−0.08	−0.06	−0.05
Client satisfaction (at post)	−0.07	−0.64	0.522	−0.19	−0.06	−0.05
IUS Residualized change	0.56	6.44	<0.001	0.54	0.52	0.51

Note. ADIS-IV = Anxiety Disorders Interview Schedule-IV, IUS = Intolerance of Uncertainty Scale.

**Table 6:** Pearson correlations among the psychological variables used in the hierarchical regression.

	Motivation (Session 1)	Working Alliance (Session 7)	Perceived Therapists’ Competence (Session 7)	Client Satisfaction (at Post)	IUS Residualized Change
ADIS-IV Residualized change	−0.07	−0.07	−0.08	−0.19 *	0.54 ***
Motivation (Session 1)		0.23 **	0.09	0.16 *	−0.08
Working alliance (Session 7)			0.66 ***	0.67 ***	−0.31 **
Perceived therapists’ competence (Session 7)				0.44 ***	−0.16
Client satisfaction (at post)					−0.38 ***

Note. ADIS-IV = Anxiety Disorders Interview Schedule-IV; IUS = Intolerance of Uncertainty Scale. * *p* < 0.05, ** *p* < 0.01, *** *p* < 0.001.

**Figure A1:**
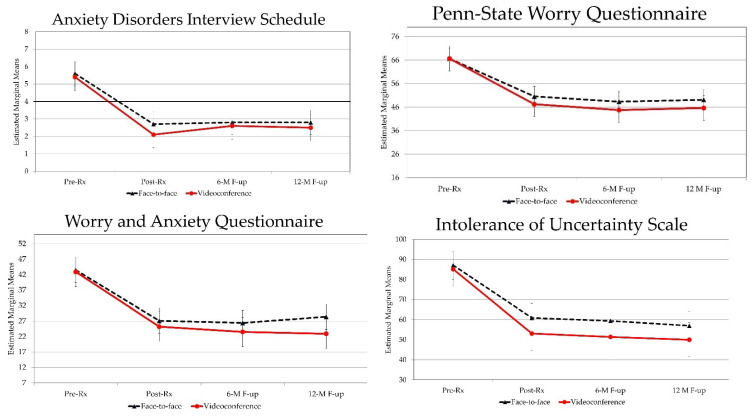

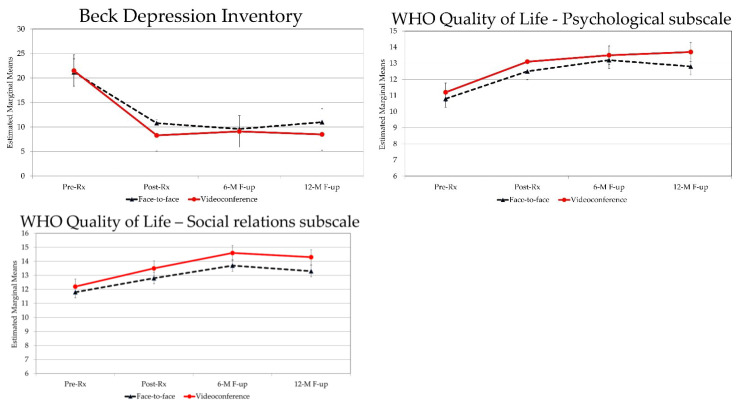
Illustration of the impact of cognitive-behavior therapy delivered by videoconference or face-to-face based on Estimated Marginal Means from Multilevel Linear Models.

## Data Availability

The dataset is not publicly available due to privacy and ethical restrictions. The data for this study are available upon request addressed directly to the Research Ethics Boards of the lead institution (comite.ethique@uqo.ca), which must first approve the request. If the request is approved, anonymized data supporting the conclusions of this manuscript will be made available by the corresponding author.

## Appendix A

Multiple linear mixed (MLM) models were also used to conduct the outcome analyses leading to the non-inferiority analyses, as alternatives to more traditional general linear model repeated measures ANOVAs. The study does not address a multilevel research question [103], but approaching the analyses from the standpoint of multilevel data allows modeling the data without forcing the covariance matrix to sphericity (compound symmetry; i.e., not all participants in each condition are assumed to change in the same way over time and to have similar slopes). MLM also handles missing data with iterative estimation based on available information in the dataset instead of removing participants from the analysis, providing an alternative to intent-to-treat and completers analyses.

The MIXED linear procedure in SPSS 28 was used, with participants’ ID as a second-level random effect and Time as a first-level fixed effect with residuals correlated within participants. An unstructured repeated covariance matrix was used for the final analyses, but a first-order autoregressive structure with homogenous variances and a diagonal structure were also tested and were all compared to a compound symmetry structure. The unstructured covariance matrix was theoretically better and provided a better fit to the data based on the AIC and BIC criteria, compared to the alternatives. Models were estimated using restricted maximum likelihood and Satterhwaite approximation. Estimated Marginal Means were computed based on the fitted model of each variable to produce the results illustrated in Figure A1.

Results of Mixed Linear Models with Condition (2) by Time (4) fixed main effects and random intercept for each participant are reported in Table 1. As the study focused on potential differences in treatment modalities, Condition by Time interaction contrasts are reported comparing end-points with pre-treatment. Results closely mirror those reported in the article, with very large treatment effects, and interactions that are not statistically significant and in the same magnitude of those reported in the more traditional analyses.

**Table A1:** Results of multilevel linear modeling analyses for a randomized control non-inferiority trial comparing the delivery of psychotherapy by videoconference or face-to-face to adults with generalized anxiety disorder (*n* = 148).

Variable	Multi Linear Modeling Tests of Fixed Effects					
	Time F	Condition F	Interaction F	Interaction Contrasts		
				Pre/post	Pre/6-Mo F-up	Pre/12-Mo F-up
ADIS	135.519 ***	2.762	0.519	1.113	0.007	0.012
PSWQ	114.037 ***	3.436	0.938	2.1	1.818	1.192
WAQ	129.011 ***	3.564	1.969	2.99	1.104	3.718
IUS	95.193 ***	5.104 *	0.857	1.66	1.775	0.676
BDI-II	60.481 ***	0.886	1.626	2.097	0.391	1.151
QOL-Psychol	43.106 ***	1.962	1.081	0.521	0.014	0.7
QOL-Social	13.991 ***	2.113	0.269	0.224	0.252	0.072

Note. ADIS = Anxiety Disorders Interview Schedule for DSM-IV, PSWQ = Penn-State Worry Questionnaire, WAQ = Worry and Anxiety Questionnaire, IUS = Intolerance of Uncertainty Scale, BDI-II = Beck Depression Inventory-II, QOL Psycho = WHO-QOL-Psychological subscale, QOL-Social = WHO-QOL-Social relations subscale. * *p* < 0.5, *** *p* < 0.001.
