# Correction: Bouchard et al. A Multisite Non-Inferiority Randomized Controlled Trial of the Efficacy of Cognitive-Behavior Therapy for Generalized Anxiety Disorder Delivered by Videoconference. *J. Clin. Med.* 2022, *11*, 5924

**DOI:** 10.3390/jcm14010226

**Published:** 2025-01-03

**Authors:** Stéphane Bouchard, Michel J. Dugas, Geneviève Belleville, Frédéric Langlois, Patrick Gosselin, Geneviève Robillard, Giulia Corno, André Marchand

**Affiliations:** 1 Département de Psychologie et de Psychoéducation, Université du Québec en Outaouais, Gatineau, QC J8X 3X7, Canada; michel.dugas@uqo.ca (M.J.D.); g.robillard@invirtuo.com (G.R.); giulia.me.corno@gmail.com (G.C.); 2 Centre de Recherche du Centre de Santé et des Services Sociaux de l’Outaouais, Gatineau, QC J8T 4J3, Canada; 3 École de Psychologie, Université Laval, Québec, QC G1V 0A6, Canada; genevieve.belleville@psy.ulaval.ca; 4 Département de Psychologie, Université du Québec à Trois-Rivières, Trois-Rivières, QC G8Z 4M3, Canada; frederic.langlois@uqtr.ca; 5 Département de Psychologie, Université de Sherbrooke, Sherbrooke, QC J1K 2R1, Canada; patrick.gosselin@usherbrooke.ca; 6 Département de Psychologie, Université du Québec à Montréal, Montréal, QC H2L 2C4, Canada; marchand.andre@uqam.ca

## Error in Tables

In the original publication [1], there was a mistake in Table 1 as published. The probabilities for the Chi-Square for the variable “Taking medication” of 3.38, *p* > 0.95. The corrected Table 1 appears below. The probabilities for the Chi-Square for the variable “Taking medication” of 3.38, *p* > 0.05.

There was a mistake in Table 2 as published. The abbreviation for standard deviation for means reported in the column for the 6-month follow-up is D. The corrected Table 2 appears below. The abbreviation for standard deviation for means reported in the column for the 6-month follow-up is SD.

There are five errors in Table 5. In the IUS Residualized change line, *std Beta* should be 0.56, *t* should be 6.44, *Simple corr.* should be 0.54, *Partial corr.* should be 0.52, *Semi-Partial corr.* should be 0.51.

**Table 5:** Contribution of non-specific and specific factors of CBT for GAD when delivered by videoconference or face-to-face at the second step of a hierarchical regression predicting improvements in ADIS-IV ratings.

	*std Beta*	*t*	*sig. p*	*Simple corr.*	*Partial corr.*	*Semi-Partial corr.*
Treatment condition	−0.12	−1.6	0.118	−0.11	−0.15	−0.12
Center providing psychotherapy	−0.03	−0.45	0.657	0.04	−0.04	−0.03
Motivation (Session 1)	−0.05	−0.67	0.502	−0.07	−0.06	−0.05
Working alliance (Session 7)	0.21	1.71	0.087	−0.07	0.16	0.13
Perceived therapists’ competence (Session 7)	−0.06	−0.65	0.52	−0.08	−0.06	−0.05
Client satisfaction (at post)	−0.07	−0.64	0.522	−0.19	−0.06	−0.05
IUS Residualized change	0.56	6.44	<0.001	0.54	0.52	0.51

Note. ADIS-IV = Anxiety Disorders Interview Schedule-IV, IUS = Intolerance of Uncertainty Scale.

There are four errors in Table 6. In the IUS Residualized Change row, ADIS-IV Residualized change should be 0.54, Working alliance (Session 7) should be −0.31, Perceived therapists’ competence (Session 7) should be −0.16, Client satisfaction (at post) should be −0.38.

**Table 6:** Pearson correlations among the psychological variables used in the hierarchical regression.

	Motivation (Session 1)	Working Alliance (Session 7)	Perceived Therapists’ Competence (Session 7)	Client Satisfaction (at Post)	IUS Residualized Change
ADIS-IV Residualized change	−0.07	−0.07	−0.08	−0.19 *	0.54 ***
Motivation (Session 1)		0.23 **	0.09	0.16 *	−0.08
Working alliance (Session 7)			0.66 ***	0.67 ***	−0.31 **
Perceived therapists’ competence (Session 7)				0.44 ***	−0.16
Client satisfaction (at post)					−0.38 ***

Note. ADIS-IV = Anxiety Disorders Interview Schedule-IV; IUS = Intolerance of Uncertainty Scale. * *p* < 0.05, ** *p* < 0.01, *** *p* < 0.001.

## Text Correction

There was an error in the original publication [1]. When reporting results from the regression in Section 3.4, the information was based on the wrong section in the output from our statistical analyses. The final regression model was statistically significant (F(7,112) = 10.05, *p* < 0.001, *R*^2^ = 0.62, Adjusted *R*^2^ = 0.35). The second step in the hierarchical regression significantly contributed to the final model (F change (1,113) = 59.4, *p* < 0.001, change in *R*^2^ = 0.32). A correction has been made to Section 3.4, end of the first paragraph: The final regression model was statistically significant (F(7,113) = 7.33, *p* < 0.001, *R*^2^ = 0.32, Adjusted *R*^2^ = 0.27). The second step in the hierarchical regression significantly contributed to the final model (F change (1,113) = 41.48, *p* < 0.001, change in *R*^2^ = 0.25).

### 3.4. Predictors Change in GAD Severity at Posttreatment

Factors potentially associated with treatment efficacy, as measured by the residualized change in ADIS-IV scores from pre to post-treatment, were examined in a hierarchical regression analysis. The common therapy factors (i.e., motivation, working alliance, perceived therapist competence and client satisfaction) were entered in the first step of the regression. Treatment condition (VCP or face-to-face therapy) and psychotherapist treatment site were also entered as methodological controls. The factor specific to CBT-IU, change in intolerance of uncertainty, was entered in the second step to test its contribution to the regression model over and above the factors entered in the first step. The final regression model was statistically significant (F(7,113) = 7.33, *p* < 0.001, *R*^2^ = 0.32, Adjusted *R*^2^ = 0.27). The second step in the hierarchical regression significantly contributed to the final model (F change (1,113) = 41.48, *p* < 0.001, change in *R*^2^ = 0.25). Table 5 details the contribution of each variable to the final model. Consistent with the non-inferiority finding for the Time by Condition interaction with the IUS, testing the direct impact of treatment conditions on the residualized change in intolerance of uncertainty was not statistically significant (F change (1,112) = 0.031, *p* = 0.86, change in *R^2^* = 0.00) and did not reduce the significant role of intolerance of uncertainty in the regression (*t* = 6.99, *p* < 0.001, semi-partial correlation = 0.52). To support the discussion of the findings, Table 6 shows the correlation among the various measures used in the regression. In the hierarchical regression, the role of sex, age, income, education, living alone, medication use, previous psychotherapy and the presence of at least one comorbid disorder were also explored. None of the aforementioned variables significantly predicted outcome or changed the conclusions of the regression analysis.

In addition, in the last paragraph of Section 1, “[54], p.16” should be changed to “[53], p.16”. The authors state that the scientific conclusions are unaffected. This Correction was approved by the Academic Editor. The original publication has also been updated.

## Figures and Tables

**Table 1:** Descriptive statistics of the intent-to-treat sample of participants diagnosed with generalized anxiety disorder who received cognitive-behavior therapy either by videoconference (VCP) or face-to-face (FF).

	VCP (*n* = 69)	FF (*n* = 79)	Statistical Test (Chi-Square or *t* Test)
Age, mean (SD)	41.35 (14.80)	39.38 (16.23)	−0.77, *p* > 0.05
Sex (female)	57 (82.60%)	65 (82.30%)	0.003, *p* > 0.05
Presence of at least one comorbid disorder *	36 (52.2%)	44 (55.7%)	0.184, *p* > 0.05
Living alone	15 (21.70%)	8 (10.10%)	3.784, *p* > 0.05
Education High school	12 (17.40%)	13 (16.50%)	0.235, *p* > 0.05
College	21 (30.40%)	27 (34.20%)	
University	36 (52.20%)	39 (49.40%)	
Work status Full-time (35 h or +)	23 (33.30%)	26 (32.90%)	3.694, *p* > 0.05
Part-time (less than 35 h)	23 (33.30%)	23 (29.10%)	
Retirement	9 (13.00%)	11 (13.90%)	
Unemployment	11 (15.90%)	9 (11.4%)	
Other	3 (4.30%)	10 (12.70%)	
Annual income Lower than 29,999$	14 (20.90%)	27 (35.10%)	4.628, *p* > 0.05
(3 refused to answer) 30 k–59,999$	25 (37.30%)	19 (29.70%)	
60 k–89,999$	11 (16.40%)	14 (18.20%)	
90 k and more	17 (25.40%)	17 (22.10%)	
Taking medication	32 (46.4%)	25 (31.6%)	3.38, *p* > 0.05
Previous psychotherapy	49 (71.00%)	57 (72.20%)	0.23, *p* > 0.05
Motivation toward therapy (Session 1)	12.38 (4.60)	12.70 (3.67)	0.46, *p* > 0.05
Working alliance (Session 7)	233.18 (18.05)	225.80 (17.52)	−2.36, *p* < 0.05
Perception of therapist competence (Session 7)	164.00 (12.99)	156.47 (19.32)	−2.49, *p* < 0.05
Client Satisfaction (post-treatment)	28.32 (3.78)	27.77 (3.46)	−0.92, *p* > 0.05

Note. VCP = Videoconference psychotherapy; FF = Face-to-face psychotherapy; SD = Standard deviation. * Participants reported having up to four comorbid conditions and the number specific comorbid conditions were as follows: social anxiety disorder (*n* = 34), panic disorder (*n* = 20), agoraphobia (*n* = 14), major depressive disorder (*n* = 14), specific phobia (*n* = 14), obsessive-compulsive disorder or trichotillomania (*n* = 7), posttraumatic stress disorder (*n* = 4), other mood disorders (*n* = 7), eating disorder (*n* = 1), other (*n* = 6).

**Table 2:** Descriptive statistics for variables used in the non-inferiority analyses (intent-to-treat) (*n* = 148).

Variable	Condition	Pre		Post		6-Month F-Up		12-Month F-Up	
		M	SD	M	SD	M	SD	M	SD
ADIS	VCP	5.41	1.07	2.96	1.90	3.25	1.78	3.25	1.78
	FF	5.62	0.90	3.15	1.95	3.17	1.90	3.19	1.85
PSWQ	VCP	66.59	7.27	51.51	11.99	49.27	12.50	49.86	12.37
	FF	66.62	7.31	53.62	11.93	51.92	13.00	52.44	12.73
WAQ	VCP	42.85	6.46	29.87	12.84	28.09	12.71	27.54	12.59
	FF	43.50	6.25	30.20	12.96	29.54	12.06	30.99	11.62
IUS	VCP	85.13	20.54	61.96	23.71	59.51	22.87	58.68	23.20
	FF	87.07	19.33	65.30	22.24	64.34	23.38	62.80	22.58
BDI-II	VCP	21.52	10.93	12.61	11.03	12.68	10.22	12.29	11.03
	FF	21.16	8.96	13.13	11.67	12.19	11.37	13.19	11.03
QOL-Psychol	VCP	11.07	2.23	12.50	2.46	12.65	2.90	12.94	2.95
	FF	10.82	2.20	12.09	2.52	12.66	3.03	12.36	2.87
QOL-Social	VCP	12.22	3.16	13.17	3.44	13.90	3.64	13.80	3.85
	FF	11.84	2.87	12.61	3.11	13.20	3.39	13.19	3.10

Note. M = Mean; SD = Standard deviation; VCP = Videoconference Psychotherapy; FF = Face-to-face; ADIS = Anxiety Disorders Interview Schedule for DSM-IV, PSWQ = Penn-State Worry Questionnaire, WAQ = Worry and Anxiety Questionnaire, IUS = Intolerance of Uncertainty Scale, BDI-II = Beck Depression Inventory-II, QOL-Psychol = WHO-QOL-Psychological subscale, QOL-Social = WHO-QOL-Social relations subscale.
